# A Regulatory Circuit Orchestrated by Novel-miR-3880 Modulates Mammary Gland Development

**DOI:** 10.3389/fcell.2020.00383

**Published:** 2020-06-16

**Authors:** Yue Zhang, Jidan Liu, Wenfei Li, Fangjun Cao, Guanglin Niu, Shengyue Ji, Xiaoyan Du, Binyun Cao, Xiaopeng An

**Affiliations:** ^1^College of Animal Science and Technology, Northwest A&F University, Yangling, China; ^2^School of Life Sciences, Technical University of Munich, Freising, Germany

**Keywords:** ciRNA13761, novel-miR-3880, *ELF2*, PI3K/AKT/mTOR/S6K1 pathway, mammary gland development

## Abstract

Milk casein and triglyceride content are important production traits in goats. Studies on mechanisms in milk casein secretion and mammary gland development is essential for milk goat breeding. miRNAs play an important role in goat lactation. While novel-miR-3880 is highly expressed at goat peak lactation stage, its molecular mechanism has not been studied. The purpose of the present study was to explore the relationship between novel-miR-3880 and lactation, as well as to construct a network among novel-miR-3880, ciRNA13761, and *E74 like ETS transcription factor 2 (ELF2)*, thus further exploring their potential roles in milk components and mammary gland development. *ELF2* was previously proven to be important in cell survival and proliferation, and 3′-UTR of *ELF2* was predicted to have binding sites of novel-miR-3880. Our study found that the overexpression of novel-miR-3880 exerted anti-apoptotic and proliferative roles in GMEC, induced a boost in triglyceride synthesis, and caused a decrease in α s1-, α s2-, and β-casein, but an increase in κ-casein secretion. Furthermore, treatment in mice indicated that novel-miR-3880 could promote mammary gland development and extend the lactation period, while novel-miR-3880 expression was found to be suppressed by ciRNA13761 as a miRNA sponge. The present study explores a mechanism of triglyceride synthesis and casein secretion, and reveals a crosstalk between ciRNA13761/novel-miR-3880/*ELF2* axis and PI3K/AKT/mTOR/S6K1 pathway, to gain a better understanding of lactation traits in dairy goats.

## Introduction

Goat’s milk is regarded as a hypoallergenic milk type with therapeutic functions and is accepted as a major functional food (Roncada et al., 2002) when casein, including κ-, β-, αs1,- and αs2-casein, is the main protein in goat’s milk (Singh and Singh, 1980; Chatchatee et al., 2001). MicroRNAs (miRNAs) are a class of endogenous non-coding RNAs of approximately 23 nt in length. They are reported to regulate gene expression by repressing translation or facilitating mRNA degradation (Ambros, 2004) and play an important role in goat lactation (Ji et al., 2017). Novel-miR-3880 was highly expressed during goat peak lactation period based on our previous sequencing data (Hou et al., 2017). The novel-miR-3880 expression data was selected from Supplementary File S1 (Hou et al., 2017). Our study explored effects of novel-miR-3880-related molecules on mammary gland development *in vivo* and *in vitro*, thus providing a theoretical molecular basis for breeding and breast care.

Our study found that the overexpression of novel-miR-3880 induced anti-apoptotic and proliferative effects, promoted triglyceride synthesis, and caused a decrease in α s1-, α s2-, and β-casein but an increase in κ-casein secretion. Further, the regulation pattern was studied. Firstly, negative control (NC) and novel-miR-3880 were transfected into goat mammary epithelial cell (MEC) and RT-qPCR was applied to detect the expression of miRNAs, thus ensuring the efficiency of RNA sequencing. Differentially expressed genes (DEGs) of novel-miR-3880 were screened, and 67 downstream genes were found. *E74 like ETS transcription factor 2* (*ELF2*), which is proven to be important in cell survival and proliferation (Qiu et al., 2008), might be essential for mammary gland development and lactation, and was found in the downregulated DEG list of sequencing results.

CircRNAs are another class of endogenous non-coding RNAs from non-classical alternative splicing that can expropriate miRNAs as a sponge to block miRNAs from binding to target genes (Hansen et al., 2013; Vicens and Westhof, 2014; Meng et al., 2017). To explore how novel-miR-3880 expression was regulated, circRNAs that have binding sites of novel-miR-3880 were screened according to circRNA sequencing data our laboratory acquired before. CircRNA13761 (ciRNA13761), whose structure is shown in Supplementary File S2, was selected and verified as a novel-miR-3880 sponge, while its source gene *DOCK1* is reported to be involved in mammary gland involution (Bagci et al., 2014). Then, the network among ciRNA13761, novel-miR-3880, and *ELF2* was constructed, and the role of *DOCK1* in the network was detected.

As is known, mTOR is a central modulator in protein/lipid synthesis and cell growth processes and plays important roles in milk production (Osorio et al., 2016; Saxton and Sabatini, 2017). It serves as a crucial downstream signal of PI3K/AKT pathway to form a functional compound (Yang et al., 2014), which participates in lactation initiation (Chen et al., 2012). S6K1, a downstream effector of mTOR, is also critical for promoting protein and lipid synthesis (Yang et al., 2014); its activation relies on phosphorylation mediated by mTOR (Magnuson et al., 2012). Our study explored whether and how novel-miR-3880 regulates PI3K/AKT/mTOR/S6K1 pathway and participates in MEC biological processes and mammary gland development. In addition, MEC anti-apoptosis signaling was evaluated by the protein expression ratio of Bcl-2 and Bax, which is regarded as a cell survival signal (Basu and Haldar, 1998).

In this study, roles of ciRNA13761, novel-miR-3880, and *ELF2* on mammary gland development and lactation traits were studied to provide a basis for molecular breeding of dairy goats. More precisely, the interaction among ciRNA13761, novel-miR-3880, and *ELF2*, and their effects on MEC triglyceride synthesis, lipid formation, casein secretion, viability, proliferation, and apoptosis were explored, as well as their participation in PI3K/AKT/mTOR/S6K1 pathway and Bcl-2/Bax pathway. Novel-miR-3880 and si*ELF2* were injected into C57BL/6 mice through the tail vein to examine the participation of PI3K/AKT/mTOR/S6K1 pathway *in vivo* and to observe the development of mammary glands affected by novel-miR-3880 and si*ELF2* with ultramicroscopic technique, to judge the availability of the molecular experiments and provide a theoretical basis for practice in dairy goat breeding and breast care.

## Materials and Methods

### Animals and Ethics

Three-year old female Guanzhong dairy goats 90-day postpartum (peak lactation period) in a research-animal-keeping farm near Northwest A&F University of Shaanxi province in China were selected and anesthetized. Then, one cubic centimeter of mammary gland tissue was removed to PBS with penicillin/streptomycin (100 U/mL, Harbin Pharmaceutical Group, China) from the middle part of the mammary gland with a scalpel. The mammary gland tissue was used to isolate MECs. The wound was sewn and sterilized immediately and animals recovered after the surgical line was removed a week later. C57BL/6 mice used in this study were of a similar age, weight, parity, and litter size, and delivered newborns on the same day. The mice were raised in an SPF environment with natural drink and food in separate nests. Each group had six nests of mice. Injective novel-miR-3880 agomir and si*ELF2* with 2′OMe and 5′Chol *in vivo* modification were bought from Ribobio (Guangzhou, China). All surgical procedures conformed to institutional and national guidelines and were approved by the Animal Care and Use Committee of the Northwest A&F University (China).

### Cell Culture and Cell Treatment

A previous method (Wang et al., 2010) was used to isolate goat MECs. Mammary epithelial cells were cultured in a basic DMEM/F12 medium (Hyclone, United States) with 10% bovine serum albumin (Gibco, United States) and penicillin/streptomycin, and incubated in 5% CO_2_ at 37°C in a humid atmosphere. Novel-miR-3880, si*ELF2*, si*DOCK1*, and si-ciRNA13761 were synthesized at GenePharma Corporation (Shanghai, China), and the sequences were GGUCCCGCCGCCGCCGCC, CCUAC CUGCUUGAGAGAUU, GCUUCGUACAUCCAUCUUA, and CCUGCACAAGGAAUGUGAU. mammary epithelial cells transfection was performed with Lipofectamine 2000 reagent (Invitrogen, United States). PI3K inhibitor (TGX-221, Selleck, Shanghai, China) in 20 μM, AKT (GDC-0068, Selleck, Shanghai, China) inhibitor in 50 μM, mTOR (Everolimus RAD001, Selleck, Shanghai, China) inhibitor in 0.5 nM, and S6K1 inhibitor (WAY-600, Selleck, Shanghai, China) in 50 μM applied to treat MEC were dissolved in DMSO, and equal DMSO was applied in the control group. The concentration was selected as suggested.

### Isolation and Analysis of RNA

Total RNA was isolated from MECs by Trizol Reagent (Invitrogen, United States) according to the manufacturer’s protocol. miRcute Plus miRNA First-Strand cDNA Kit (Tiangen, Beijing, China) was used to perform reverse transcription of novel-miR-3880. The PrimeScript RT Reagent Kit with gDNA Eraser (Takara, Japan) was used to acquire cDNA. Oligo dT primer was applied for mRNA reverse transcription; for cDNA of ciRNA, total RNA was dealt with RNase, and then random primer was used for reverse transcription. RT-qPCR was conducted using SYBR Green qPCR Master Mix (Takara, Japan) to analyze the mRNA, miRNA, or ciRNA expression levels. Primers used in RT-qPCR are shown below. *ELF2*: TGTGGCGGTTCAGTCAGTTA (forward), CAGTAGAGGCTGGCATCACA (reverse); *Dock1*: CGGGA CTCAGAACTCATCGG (forward), CCACCACATCGGTCC TCATC (reverse); ciRNA13761: GAAGTGGTCGGAGGACGTG (forward); TTGTGCAGGTCACAGAGCTT (reverse); β*-actin*: GATCTGGCACCACACCTTCT (forward); GGGTCATCTTC TCACGGTTG (reverse); novel-miR-3880: TATATAGCCGCCG CCGCC (forward); *U6*: CTCGCTTCGGCAGCACA (forward); AACGCTTCACGAATTTGCGT (reverse).

### RNA-Sequencing

Goat MEC RNA quality was analyzed with an Agilent bioanalyzer 2100, and mRNA was captured using the Poly (A) mRNA Magnetic Isolation Module (NEBNext, United States). The library was constructed using an Ultra RNA Library Prep Kit for Illumina (NEBNext, United States). After the library was purified by Agencourt AMPure XP beads (Beckman, United States), the quality of the library was detected again using an Agilent Bioanalyzer 2100 and Qubit. In addition, before Illumina HiSeq, cBOT automatic clustering was conducted using the TruSeq PE Cluster Kit v4.

### Vector Construction

PsiCHECK-2 empty vector was purchased from Promega (Madison, United States). *ELF2*-1 3′UTR and *ELF2*-2 3′UTR containing the seed site were amplified and inserted into psiCHECK-2 vector as *ELF2*-1 and *ELF2*-2 wild type (Wt) vectors. Primers for *ELF2*-1 3′UTR were CTAGGGTGTTAGTGCCGGTC (forward) and CGACAAGATCACCCATCCCA (reverse); for *ELF2*-2 3′UTR were TTCCCAACTGCTGCGTGAA (forward) and GAGTTACAGGACCTAGTTTGGTGT (reverse). The vectors sequencing documents are shown in Supplementarys Files S3, S4. *ELF2*-1 Mutant (Mu) and ciRNA13761 Wt/Mu psiCHECK-2 vectors were provided by Tsingke Biological Technology Company (Beijing, China).

The complete CDS region of *ELF2* was amplified with primers ATGACATCAGCAGTGGTTGAC (forward) and TCATTTCTCACACGCTACCAG (reverse) and inserted into pcDNA3.1(+)vector. The sequencing result of pcDNA3.1-*ELF2* vector is provided in Supplementary File S5. In addition, ciRNA13761-pcDNA3.1(+) CircRNA Mini Vector was provided by Tsingke Biological Technology Company (Beijing, China) and marked as pcDNA3.1^#^-ciRNA13761.

### Dual-Luciferase Reporter Assays

Luciferase activities were measured in accordance with the protocol of Dual-Luciferase Reporter Assay System (Promega, Madison, WI, United States). The ratio of *hRluc* and *hluc*^+^ activities was used to calculate the relative luciferase activity.

### Western Blot Analysis

Equal amounts of each protein sample was loaded to detect protein expression. The following primary antibodies were used in this experiment: β-actin (Beyotime, AA128, Shanghai, China), ELF2 (Proteintech, 12499-1-AP, United States), Bax (BBI, D220073, Shanghai, China), Bcl-2 (BBI, D260117, Shanghai, China), PI3K p110 beta (Bioss, bs-6423R, Beijing, China), p-PI3K p110 beta (Bioss, bs-6417R, Beijing, China), AKT (Cell Signaling Technology, 4685, United States), p-AKT (Cell Signaling Technology, 4060, United States), mTOR (Boster Biological Technology, BM4182, United States), and p-mTOR (Boster Biological Technology, BM4840, United States). Horse Radish Peroxidase-conjugated goat anti-rabbit IgG secondary antibodies were purchased from Beyotime (Shanghai, China).

### Immunohistochemistry

Slides cut from paraffin-embedded tissues underwent drying, rehydration, antigen retrieval, and permeation before the samples were blocked in goat serum for 20 min and incubated in the primary antibody then in the second antibody. Color development was performed with a DAB Substrate kit (Solarbio, DA1010, Beijing, China) and counterstained with Hematoxylin (Solarbio, H8070, Beijing, China).

### Flow Cytometry

MEC apoptotic rate was evaluated using the Annexin V-FITC/PI apoptosis kit (7Sea Biotech, Shanghai, China) and MEC cell cycle was measured by Cell cycle staining Kit (MultiSciences, Hangzhou, China) according to the manufacturer’s instructions.

### Triglyceride Content Detection and Oil Red O Staining

Triglyceride content was measured by Tissue/Cell triglyceride assay kit (Applygen, Beijing, China) and normalized to protein concentration, which was tested by BCA protein assay kit. Oil red O used in staining was purchased from Solarbio (Beijing, China). Relative density of lipid drops was analyzed by Image-Pro Plus 6.0 Software.

### ELISA

MEC cultivator was acquired to detect the contents of α s1-, α s2-, β-, and κ-casein respectively using casein ELISA Kits specialized for goats (mlbio, Shanghai).

### Cell Counting Kit-8 (CCK-8) Assay

Ten microliter of CCK-8 solution (7Sea Biotech, Shanghai, China) was added into MECs seeded in 96-well plates and incubated at 37°C for 4 h to evaluate MEC viability. Then, the optical density value was measured at 450 nm with a Microplate Reader (Bio Tek, United States).

### 5-Ethynyl-2′-Deoxyuridine (EdU) Assay

EdU fluorescence labeling was achieved by EdU Cell Proliferation Kit with Alexa Fluor 488 (Beyotime, Shanghai, China), and cell nucleuses were dyed with DAPI (Beyotime, Shanghai, China).

### Statistical Analysis

Sequencing data quality was evaluated using FastQC v0.10.1 and data filtering was performed with Cutadapt 1.9.1. Short read alignment was conducted using Hisat v2.0.14. SNV and InDel information was acquired from Samtools v0.1.18 analysis. The weight of female offspring in every nest was averaged and the data in six nests were set as one group. The acquired data of three groups were applied to analyze. Experiments in this study were conducted in three independent repeats. SPSS 22.0 was used to conduct one-way ANOVA and Student *t*-test. ^∗^*p* < 0.05, which was considered significant, and ^∗∗^*p* < 0.01, which was considered highly significant.

## Results

### Novel-miR-3880 Directly Suppresses the Expression of *ELF2*

**FIGURE 1:**
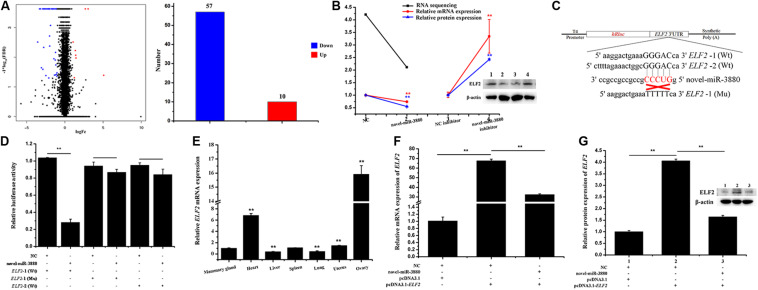
Confirmation of the relationship between novel-miR-3880 and *ELF2*. **(A)** DEG number acquired from RNA sequencing. Dots in region logFc < 0 represent downregulated genes, otherwise, in region logFc > 0 means upregulated genes. In total, there are 57 downregulated DEGs and 10 upregulated DEGs. **(B)** Novel-miR-3880 suppressed *ELF2* expression in RNA sequencing, mRNA, and protein levels. When novel-miR-3880 was inhibited, mRNA and protein levels were improved. **(C)** Schematic diagram explaining the design of *ELF2*-1 (Wt), *ELF2*-2 (Wt) and *ELF2*-1 (Mu) luciferase reporter vectors. **(D)** *ELF2*-1 (Wt), *ELF2*-2 (Wt) and *ELF2*-1 (Mu) reporter vectors were co-transfected with novel-miR-3880 or NC into MEC, and luciferase assay was performed at 48 h after transfection. **(E)** Goat tissue expression profile of *ELF2*. **(F,G)** *ELF2* overexpression vector pcDNA3.1-*ELF2* effectiveness was verified in mRNA and protein levels. Novel-miR-3880 significantly decreased the efficiency of pcDNA3.1-*ELF2* vector.

After novel-miR-3880 was transfected into MECs with NC as control, RNA sequencing was conducted and DEGs are shown in Supplementary File S6. There were 57 downregulated DEGs and 10 upregulated DEGs in novel-miR-3880 group (Figure 1A). *ELF2* selected from DEGs of RNA-sequencing was verified via RT-qPCR and western blotting to evaluate the sequencing accuracy (Figure 1B).

A schematic diagram of dual luciferase reporter vectors was described in Figure 1C. It is shown in Figure 1D that the relative luciferase activity was lowered when novel-miR-3880 was co-transfected with psiCHECK2-*ELF2*-1 3′-UTR Wt vector. However, the relative luciferase activity stayed the same level when either NC or novel-miR-3880 were co-transfected with *ELF2*-2 3′-UTR Wt vector or psiCHECK2-*ELF2*-1 3′-UTR Mu vector (Figure 1D). In addition, the expression of *ELF2* in different goat tissues was detected (Figure 1E) and pcDNA3.1-*ELF2* overexpression vector efficiency is provided in Figures 1F,G.

### CiRNA13761 Sponges Novel-miR-3880 and Induces a Regulation in ciRNA13761/Novel-miR-3880/*ELF2* Network

**FIGURE 2:**
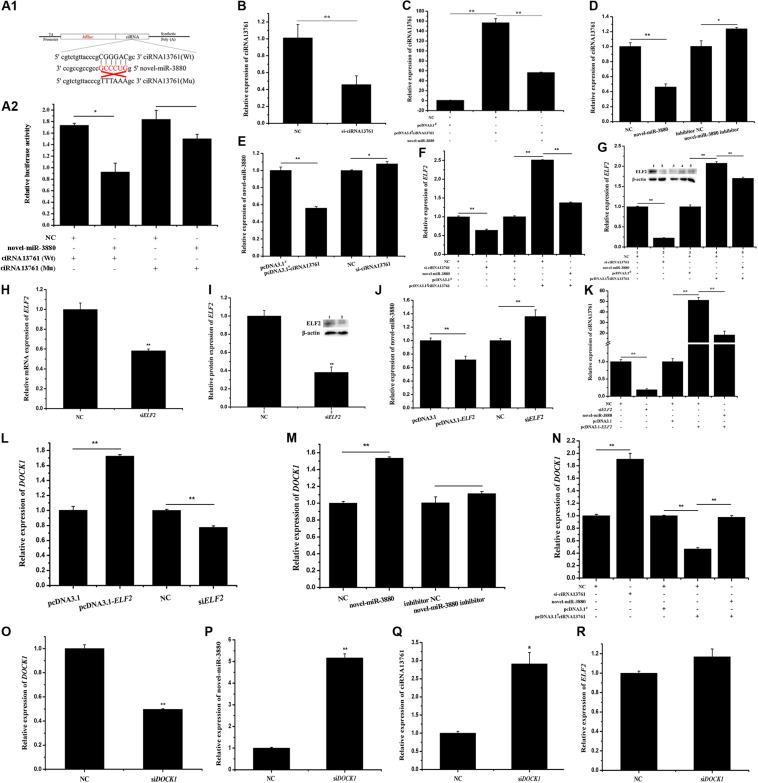
Network construction of ciRNA13761/*DOCK1*/novel-miR-3880/*ELF2* axis. **(A)** Information of Wt and Mu psiCHECK2-ciRNA13761 Wt and Mu vectors design and relative luciferase activity when vectors were co-transfected with novel-miR-3880 or NC. **(B)** The efficiency of si-ciRNA13761. **(C)** The efficiency of pcDNA3.1^#^-ciRNA13761 and effects of novel-miR-3880 on pcDNA3.1^#^- ciRNA13761 efficiency. **(D)** Effects of novel-miR-3880 on ciRNA13761 expression. **(E)** The regulation of ciRNA13761 to novel-miR-3880 expression. **(F,G)** The regulation of ciRNA13761 and novel-miR-3880 to *ELF2* mRNA and protein. **(H,I)** The efficiency of si*ELF2* at mRNA and protein levels. **(J,K)** Effects of *ELF2* on ciRNA13761 and novel-miR-3880 expression; the existence of novel-miR-3880 could impair the expression of ciRNA13761 enhanced by *ELF2* overexpression. **(L–N)** Effects of *ELF2*, novel-miR-3880 and ciRNA13761 on *DOCK1* expression. **(O)** The efficiency of si*DOCK1*. **(P–R)** The regulation of si*DOCK1* to *ELF2*, novel-miR-3880 and ciRNA13761.

Relative luciferase activity shown in Figure 2A illustrates that the relative luciferase activity of Wt psiCHECK2-ciRNA13761 vector was reduced, but that of the Mu vector was not changed, which indicates novel-miR-3880 was absorbed by ciRNA13761. To explore the regulatory pattern of ciRNA13761, the network among ciRNA13761, novel-miR-3880, and *ELF2* was established, and the role of ciRNA13761 source gene-*DOCK1* in the network was determined. The efficiency of si- and pcDNA3.1^#^-ciRNA13761 is clarified in Figures 2B,C; it could be seen in Figures 2C,D that novel-miR-3880 decreased the expression of ciRNA13761. Combined with the effect of ciRNA13761 on novel-miR-3880 shown in Figure 2E, it showed a mutual inhibition between novel-miR-3880 and ciRNA13761. Furthermore, ciRNA13761 induced an increase in *ELF2* expression, while si-ciRNA13761 decreased *ELF2* expression (Figures 2F,G). To determine the function of *ELF2* in the network, the efficiency of si*ELF2* was verified (Figures 2H,I). It is evident that *ELF2* inhibited novel-miR-3880 (Figure 2J), but promoted ciRNA13761 and *DOCK1* expression (Figures 2K,L). Otherwise, novel-miR-3880 improved but ciRNA13761 restrained the expression of *DOCK1* (Figures 2M,N). Interestingly, as *DOCK1* was knocked down (Figure 2O), novel-miR-3880 and ciRNA13761 expression were enhanced while *ELF2* expression was not affected (Figures 2P,R).

### Novel-miR-3880 Improves MEC Viability and Suppresses Apoptosis via *ELF2*

As CCK-8 results revealed, si-ciRNA13761, novel-miR-3880, and si*ELF2* improved MEC viability (Supplementary File S7a) while novel-miR-3880 recovered MEC viability reduction caused by ciRNA13761 and *ELF2* overexpression (Supplementary Files S7b,c). Meanwhile, late apoptotic rates of MEC were analyzed and found the apoptosis of MEC was restrained by si-ciRNA13761, novel-miR-3880, and si*ELF2*, while rising the apoptotic rate induced by ciRNA13761. *ELF2* overexpression was balanced by novel-miR-3880 (Supplementary Files S7d–f). MEC cell cycle did not change significantly when the expression of novel-miR-3880 was overexpressed (Supplementary File S7g).

### CiRNA13761/Novel-miR-3880/*ELF2* Axis Activates PI3K/AKT/mTOR/S6K1 Pathway

**FIGURE 3:**
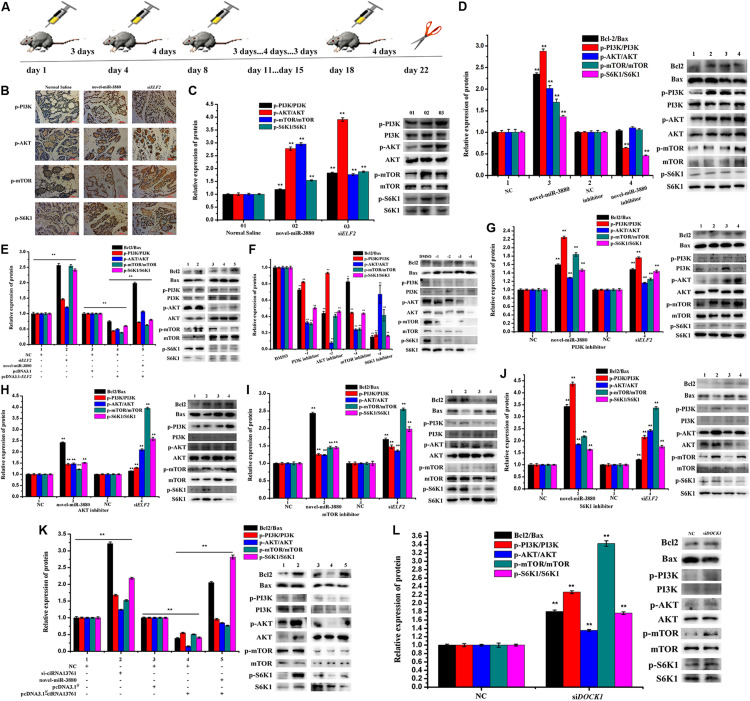
Relationship between ciRNA13761/novel-miR-3880/*ELF2* axis and PI3K/AKT/mTOR/S6K1 pathway. **(A)** Schematic diagram of animal treatment. C57BL/6 mice were injected with novel-miR-3880 or si*ELF2* in an interval of three days and four days alternatively. Samples were harvested at day 22. **(B)** Immunohistochemistry of mouse mammary gland for p-PI3K, p-AKT, p-mTOR and p-S6K1 in Normal Saline, novel-miR-3880 and si*ELF2* groups. **(C)** Protein phosphorylation level of PI3K, AKT, mTOR and S6K1 in mouse mammary gland. **(D,E)** Effects of novel-miR-3880 and *ELF2* on Bcl2/Bax pathway and protein phosphorylation level of PI3K, AKT, mTOR and S6K1 in MEC. **(F)** PI3K, AKT, mTOR and S6K1 inhibitors suppressed the phosphorylation of PI3K, AKT, mTOR and S6K1 in MEC. **(G–J)** The role of novel-miR-3880 and si*ELF2* in Bcl2/Bax and protein phosphorylation level of PI3K, AKT, mTOR and S6K1 in MEC with PI3K, AKT, mTOR or S6K1 inhibited. **(K)** Regulation of ciRNA13761 on Bcl2/Bax and PI3K, AKT, mTOR and S6K1 phosphorylation, and the balance effects of novel-miR-3880. **(L)** Effects of si*DOCK1* on Bcl2/Bax and PI3K, AKT, mTOR and S6K1 phosphorylation.

C57BL/6 mice during the lactation period were injected with novel-miR-3880 or si*ELF2* and a schematic diagram about the operational details is shown in Figure 3A. Postpartum mice were injected with 10 nmol novel-miR-3880 or si*ELF2* per individual with six mice in a group, while an interval of three days and four days was applied alternately. Mammary gland tissue and blood samples were collected at day 22. The phosphorylation levels of PI3K, AKT, mTOR, and S6K1 in mammary gland tissue were shown in Figures 3B,C, which showed a higher phosphorylation of PI3K/AKT/mTOR/S6K1 in novel-miR-3880 and si*ELF2* groups. In MEC, novel-miR-3880 and si*ELF2* enhanced the phosphorylation of PI3K, AKT, mTOR, and S6K1, and novel-miR-3880 balanced the reduction of phosphorylation caused by *ELF2* overexpression (Figures 3D,E). Then PI3K, AKT, mTOR, and S6K1 inhibitors dissolved in DMSO were applied to demonstrate the role of novel-miR-3880 and si*ELF2* in PI3K/AKT/mTOR/S6K1 pathway. In Figure 3F, PI3K, AKT, mTOR, and S6K1 inhibitors show a negative effect to the phosphorylation of each other, and the level of Bcl2/Bax was decreased when PI3K, AKT, mTOR, and S6K1 were inhibited. The results indicated that novel-miR-3880 and si*ELF2* could alleviate the reduction of phosphorylated-PI3K/AKT/mTOR/S6K1 and Bcl2/Bax levels (Figures 3G–J). However, Figures 3K,L show that ciRNA13761 played a negative role in PI3K/AKT/mTOR/S6K1 pathway activation, but PI3K/AKT/mTOR/S6K1 pathway was reactivated when novel-miR-3880 was co-transfected or ciRNA13761/*DOCK1* was knocked down.

### Novel-miR-3880 Enhances MEC Proliferation Through PI3K/AKT/mTOR/S6K1 and Bcl-2/Bax Pathway via Targeting *ELF2*

The results show that MEC proliferation would be suppressed when PI3K, AKT, mTOR, or S6K1 were inhibited (Supplementary Files S8a,b), but the participation of novel-miR-3880 and si*ELF2* played a positive part in freeing MEC to proliferate (Supplementary Files S8a,c–g). In addition, results shown in Supplementary Files S8h–k provide evidence for the restraint of ciRNA13761 and *ELF2* overexpression on MEC proliferation and the restraint was alleviated by novel-miR-3880, and further affirmed the modulation of ciRNA13761, novel-miR-3880, and *ELF2* on MEC proliferation.

### ELF2 Contributes to the Modulation of Novel-miR-3880 to MEC Lipid Formation, Triglyceride Synthesis and Casein Secretion

The Oil Red O staining and triglyceride content test revealed that novel-miR-3880, si-ciRNA13761, and si*ELF2* enhanced while ciRNA13761 and *ELF2* overexpression reduced MEC lipid formation (Supplementary Files S9a–c) and triglyceride synthesis (Supplementary Files S9d–f), with the reduction weakened by novel-miR-3880 (Supplementary Files S9b,c,e,f). The participation of PI3K/AKT/mTOR/S6K1 pathway is illustrated in Supplementary File S9g; it is shown that MEC triglyceride content declined significantly when PI3K, AKT, mTOR, or S6K1 was inhibited. It is worthwhile to mention that novel-miR-3880 and si*ELF2* improved the triglyceride content in mouse mammary glands but did not change blood triglyceride content (Supplementary Files S9h,i).

As is shown in Supplementary File S10a, novel-miR-3880, si-ciRNA13761, and si*ELF2* increased MEC κ-casein secretion, but decreased α s1-, α s2-, and β-casein secretion. The ciRNA13761 and *ELF2* overexpression and novel-miR-3880 co-transfection experiment demonstrated an interaction among ciRNA13761, novel-miR-3880, and *ELF2* in MEC κ-, α s1-, α s2-, and β-casein secretion regulation (Supplementary Files S10b,c).

### Novel-miR-3880 Promotes Mammary Gland Development, Extends Lactation Period and Benefits Offspring Growth

**FIGURE 4:**
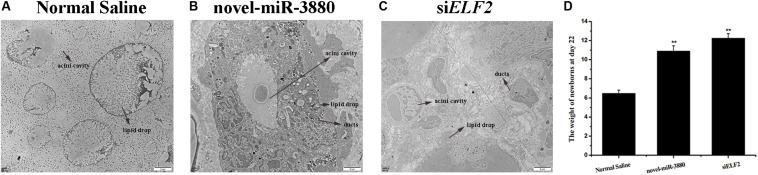
Mammary gland morphology after treatment with Normal Saline, novel-miR-3880, and si*ELF2* and weight of female offspring in each group. **(A–C)** Novel-miR-3880 and si*ELF2* promoted mammary gland development and extended lactation period. **(D)** Offspring fed by mothers in novel-miR-3880 and si*ELF2* groups grew faster than normal. There were 28 females (*n* = 4, 5, 5, 4, 6, 4) weighed in Normal Saline group, 30 females (*n* = 6, 7, 5, 4, 4, 4) in novel-miR-3880 group and 32 females (*n* = 4, 6, 5, 5, 6, 6) in si*ELF2* group.

The ultrastructure of collected mammary gland tissues was observed. It is significant that lipid droplets became larger but fewer, as the acini cavities turned smaller in the control group (Figure 4A). However, animals injected with novel-miR-3880 or si*ELF2* were still lactating with plump acini filled with secreta and had more abundant mammary ducts (Figures 4B,C). More images can be found in Supplementary File S11. The weight of all female offspring in six nests of each group was measured, and it was found that newborns fed by mothers in novel-miR-3880 or si*ELF2* groups gained more weight (Figure 4D).

## Discussion

This research aimed to study the effects of novel-miR-3880 and relative molecules on mammary gland development and lactation traits, thus gaining a better understanding of lactation in dairy goats. A general view of our work is presented in Supplementary File S12. Novel-miR-3880 was selected from our previous study (Hou et al., 2017) for its higher expression in peak lactation stage. The present study reveals that novel-miR-3880 targeted *ELF2* and was sponged by ciRNA13761. CiRNA13761, novel-miR-3880, and *ELF2* as well as *DOCK1* had an interaction with each other, although si*DOCK1* did not directly affect the expression of *ELF2*. It indicates that the molecules we studied have a mutual regulation to keep a steady state in MEC. Novel-miR-3880 combined with *ELF2* 3′-UTR to suppress the expression of *ELF2*, while ciRNA13761 sponged novel-miR-3880 and reduced the chance of combination between novel-miR-3880 and *ELF2*, and *ELF2* inhibited the expression of novel-miR-3880 to protect itself from too low expression. Interestingly, novel-miR-3880 also presented resistance to ciRNA13761 to ensure its own function in the network. *DOCK1*, as the source gene of ciRNA13761, improved both expression of novel-miR-3880 and ciRNA13761 when it was knocked down, while *ELF2* exerted a facilitated role in *DOCK1* expression. Our findings suggest that the network participates in MEC triglyceride synthesis, casein secretion, cell viability, and proliferation, as well as anti-apoptosis regulation, and plays an important part in PI3K/AKT/mTOR/S6K1 and Bcl-2/Bax pathways.

Novel-miR-3880 improved MEC triglyceride synthesis, casein secretion, cell viability, proliferation, and anti-apoptotic ability via targeting *ELF2.* Novel-miR-3880 would offset the reduction caused by ciRNA13761 and *ELF2* overexpression. This confirms the mutual regulation among ciRNA13761, novel-miR-3880, and *ELF2* on MEC. Besides, the results shown in Figure 3 not only demonstrate that ciRNA13761, novel-miR-3880, *ELF2*, and *DOCK1* engaged in PI3K/AKT/mTOR/S6K1 pathway and Bcl2/Bax signaling, but also certify a close relationship among PI3K, AKT, mTOR, and S6K. Once any of them was inhibited, all of them would face a decrease in phosphorylation level as well as a decline in the expression of Bcl2/Bax. Fortunately, novel-miR-3880 and si*ELF2* could activate PI3K/AKT/mTOR/S6K1 and Bcl2/Bax pathways, and possesses the ability to alleviate the decline of PI3K/AKT/mTOR/S6K1 phosphorylation levels and Bcl2/Bax expression ratio, which might be a reason for the mitigative effects of novel-miR-3880 and si*ELF2* on reduced MEC proliferation induced by PI3K, AKT, mTOR, or S6K1 inhibitor. It thus appears that the results are consistent with previous research that PI3K/AKT/mTOR/S6K1 pathway activation benefits cell growth (Park et al., 2011; Zhang et al., 2019), and provide sufficient evidence for the participation of novel-miR-3880 and *ELF2* in the modulation of PI3K/AKT/mTOR/S6K1 pathway both *in vitro* and *in vivo*. In addition, the role of ciRNA13761 and *DOCK1* in PI3K/AKT/mTOR/S6K1 pathway and Bcl2/Bax pathway was elaborated, which increases evidence for the regulation of ciRNA13761/novel-miR-3880/*ELF2* to PI3K/AKT/mTOR/S6K1 and Bcl2/Bax pathway.

The results provide evidence that novel-miR-3880 is a key molecule in regulating the effects of ciRNA13761 and *ELF2* on MEC function, illustrating the role of their mutual regulation in MEC cellular processes. It is worth mentioning that novel-miR-3880 decreased content of most casein (α s1-, α s2-, and β-casein), which makes it closer to human milk protein composition (Kunz and Lonnerdal, 1992; Hao-Feng, 2012), and increased κ-casein content to make milk products easier to process (Brigid et al., 2003).

Importantly, it was confirmed that novel-miR-3880 and si*ELF2* work well in facilitating mammary gland development, improving triglyceride content in mammary glands but not in blood, extending lactation days, and promoting newborn growth through PI3K/AKT/mTOR/S6K1 pathway. Therefore, novel-miR-3880 application is promising in the goat breeding and milk industry.

## Data Availability Statement

All datasets generated for this study are included in the article/Supplementary Material.

## Ethics Statement

The animal study was reviewed and approved by the Animal Care and Use Committee of Northwest A&F University.

## Author Contributions

BC and XA guided the experiment and applied the funds. YZ designed and performed most of the experiment and wrote the manuscript. FC, WL, JL, and XD conducted some of experiments. GN and SJ revised the manuscript. All of the authors have read and approved the submitted version of the manuscript.

## Conflict of Interest

The authors declare that the research was conducted in the absence of any commercial or financial relationships that could be construed as a potential conflict of interest.

## Supplementary Material

FILE S1 Differentially expressed miRNAs between Colostrum period and peak period acquired from previous sequencing results in our laboratory (Hou et al., 2017).

FILE S2 Source and structure of ciRNA13761.

FILE S3 Result of psiCHECK2-*ELF2*-1 vector sequencing.

FILE S4 Result of psiCHECK2-*ELF2*-2 vector sequencing.

FILE S5 Result of pcDNA3.1-*ELF2* vector sequencing.

FILE S6 Novel-miR-3880 regulated differentially expressed genes in MEC acquired from RNA-sequencing.

FILE S7 MEC viability and apoptosis regulated by *ELF2*, novel-miR-3880, and ciRNA13761. **(a–c)** MEC viability was regulated by novel-miR-3880, ciRNA13761 and *ELF2*. **(d–f)** The modulation of novel-miR-3880, ciRNA13761 and *ELF2* to MEC apoptosis. **(g)** The effect of novel-miR-3880 on MEC cycle.

FILE S8 Roles of PI3K/AKT/mTOR/S6K1 pathway and ciRNA13761/novel- miR-3880/*ELF2* axis in MEC proliferation. **(a–g)** In accordance with the EdU results **(a)**, PI3K, AKT, mTOR, and S6K1 innhibitors restrained MEC proliferation **(b)**, while novel-miR-3880 and si*ELF2* aroused MEC proliferation **(c)**; and novel-miR-3880 and si*ELF2* could suppress the inhibition come from PI3K **(d)**, AKT **(e)**, mTOR **(f)** and S6K1 innhibitors **(g)**. **(h,i)** Regulation of ciRNA13761 to MEC proliferation and the function of novel-miR-3880 on balancing reduced MEC proliferation caused by ciRNA13761 overexpression. **(j,k)** Regulation of *ELF2* to MEC proliferation and the balance of novel-miR-3880 on reduced MEC proliferation caused by *ELF2* overexpression.

FILE S9 Effects of ciRNA13761, novel-miR-3880, and *ELF2* on MEC lipid droplets formation and triglyceride synthesis. **(a–c)** Oil red O staining illustrating the amount of MEC lipid droplets. **(d)** MEC triglyceride content was improved by si-ciRNA13761, novel-miR-3880, si*ELF2*. **(e,f)** *ELF2* and ciRNA13761 overexpression restrained triglyceride synthesis while novel-miR-3880 eliminated some restraint. **(g)** PI3K, AKT, mTOR and S6K1 inhibitors suppressed triglyceride synthesis in MEC. **(h)** Novel-miR-3880 and si*ELF2* improved triglyceride content in mouse mammary gland. **(i)** Novel-miR-3880 and si*ELF2* did not change triglyceride content in blood of mouse.

FILE S10 Effects of ciRNA13761, novel-miR-3880, and *ELF2* on κ-, α s1-, αs2- and β-casein secretion in MEC. **(a)** Effects of si-ciRNA13761, novel-miR-3880 and si*ELF2* on casein secretion. **(b)** Regulation of overexpressed ciRNA13761 to casein secretion. **(c)** Regulation of overexpressed *ELF2* to casein secretion.

FILE S11 General view of mutual regulation among ciRNA13761, *DOCK1*, novel-miR-3880, and *ELF2*.

## References

[B1] Ambros V. (2004). The functions of animal microRNAs. *Nature* 431 350–355. 10.1038/nature02871 15372042

[B2] Bagci H. Laurin M. Huber J. Muller W. J. Côté J. F. (2014). Impaired cell death and mammary gland involution in the absence of Dock1 and Rac1 signaling. *Cell Death Dis.* 5:e1375. 10.1038/cddis.2014.338 25118935 PMC4454313

[B3] Basu A. Haldar S. (1998). The relationship between BcI2. Bax and p53: consequences for cell cycle progression and cell death. *Mol. Hum. Reprod.* 4 1099–1109. 10.1093/molehr/4.12.1099 9872359

[B4] Brigid B. Smolenski G. Wheeler T. Wells D. L’Huillier P. Laible G. (2003). Cloned transgenic cattle produce milk with higher levels of beta-casein and kappa-casein. *Nat. Biotechnol.* 21:157. 10.1038/nbt783 12548290

[B5] Chatchatee P. Järvinen K. M. Bardina L. Vila L. Beyer K. Sampson H. A. (2001). Identification of IgE and IgG binding epitopes on beta- and kappa-casein in cow’s milk allergic patients. *Clin. Exp. Allergy* 31 1256–1262. 10.1046/j.1365-2222.2001.01167.x 11529896

[B6] Chen C.-C. Stairs D. B. Boxer R. B. Belka G. K. Horseman N. D. Alvarez J. V. (2012). Autocrine prolactin induced by the Pten-Akt pathway is required for lactation initiation and provides a direct link between the Akt and Stat5 pathways. *Genes Dev.* 26 2154–2168. 10.1101/gad.197343.112 23028142 PMC3465737

[B7] Hansen T. B. Jensen T. I. Clausen B. H. Bramsen J. B. Finsen B. Damgaard C. K. (2013). Natural RNA circles function as efficient microRNA sponges. *Nature* 495 384–388. 10.1038/nature11993 23446346

[B8] Hao-Feng G. U. (2012). Comparison of nutritional components for goat milk, cow milk and human milk. *Sci. Technol. Food Indus.* 33 369–373.

[B9] Hou J. An X. Song Y. Cao B. Yang H. Zhang Z. (2017). Detection and comparison of microRNAs in the caprine mammary gland tissues of colostrum and common milk stages. *BMC Genet.* 18:38. 10.1186/s12863-017-0498-2 28464792 PMC5414302

[B10] Ji Z. Liu Z. Chao T. Hou L. Fan R. He R. (2017). Screening of miRNA profiles and construction of regulation networks in early and late lactation of dairy goat mammary glands. *Sci. Rep.* 7:11933. 10.1038/s41598-017-12297-4 PMC5607250 28931951

[B11] Kunz C. Lonnerdal B. (1992). Re-evaluation of the whey protein/casein ratio of human milk. *Acta Paediatr.* 81 107–112. 10.1111/j.1651-2227.1992.tb12184.x 1515752

[B12] Magnuson B. Ekim B. Fingar D. C. (2012). Regulation and function of ribosomal protein S6 kinase (S6K) within mTOR signalling networks. *Biochem. J.* 441 1–21. 10.1042/bj20110892 22168436

[B13] Meng X. Li X. Zhang P. Wang J. Zhou Y. Chen M. (2017). Circular RNA: an emerging key player in RNA world. *Brief. Bioinform.* 18 547–557. 27255916 10.1093/bib/bbw045

[B14] Osorio J. S. Lohakare J. Bionaz M. (2016). Biosynthesis of milk fat, protein, and lactose: roles of transcriptional and posttranscriptional regulation. *Physiol. Genomics* 48 231–256. 10.1152/physiolgenomics.00016.2015 26812986

[B15] Park K. R. Nam D. Yun H. M. Lee S. G. Jang H. J. Sethi G. (2011). beta-Caryophyllene oxide inhibits growth and induces apoptosis through the suppression of PI3K/AKT/mTOR/S6K1 pathways and ROS-mediated MAPKs activation. *Cancer Lett.* 312 178–188. 10.1016/j.canlet.2011.08.001 21924548

[B16] Qiu Y. Morii E. Zhang B. Tomita Y. Aozasa K. (2008). E74-like factor 2 transactivates valosin-containing protein gene, a gene involved in cancer growth? *Exp. Mol. Pathol.* 84 226–229. 10.1016/j.yexmp.2008.04.004 18544453

[B17] Roncada P. Gaviraghi A. Liberatori S. Canas B. Bini L. Greppi G. F. (2002). Identification of caseins in goat milk. *Proteomics* 2 723–726. 10.1002/1615-9861(200206)2:6<723::aid-prot723>3.0.co;2-i 12112854

[B18] Saxton R. A. Sabatini D. M. (2017). mTOR Signaling in Growth. Metabolism, and Disease. *Cell* 69 361–371. 10.1016/j.cell.2017.03.035 28388417

[B19] Singh V. B. Singh S. N. (1980). Total protein, whey protein and casein content milk of four Indian goat breeds during lactation. *Int. Goat Sheep Res.*

[B20] Vicens Q. Westhof E. (2014). Biogenesis of Circular RNAs. *Cell* 159 13–14. 10.1016/j.cell.2014.09.005 25259915

[B21] Wang Z. Luo J. Wang W. Zhao W. Lin X. (2010). [Characterization and culture of isolated primary dairy goat mammary gland epithelial cells]. *Sheng Wu Gong Cheng Xue Bao* 26 1123–1127. 21090118

[B22] Yang L. Miao L. Liang F. Huang H. Teng X. Li S. (2014). The mTORC1 effectors S6K1 and 4E-BP play different roles in CNS axon regeneration. *Nat. Commun.* 5:5416. 10.1038/ncomms6416 PMC4228696 25382660

[B23] Zhang Z. Zhang X. Zhao D. Liu B. Wang B. Yu W. (2019). TGFbeta1 promotes the osteoinduction of human osteoblasts via the PI3K/AKT/mTOR/S6K1 signalling pathway. *Mol. Med. Rep.* 19 3505–3518. 30896852 10.3892/mmr.2019.10051 PMC6471541

