# Supplementary material for: A Regulatory Circuit Orchestrated by Novel-miR-3880 Modulates Mammary Gland Development
Source: Front Cell Dev Biol. 2020 Jun 16;8:383. doi: 10.3389/fcell.2020.00383 (PMC7325939; doi:10.3389/fcell.2020.00383)
Supplement: FILE S1 — Differentially expressed miRNAs between Colostrum period and peak period acquired from previous sequencing results in our laboratory (Hou et al., 2017). [file Data_Sheet_1.docx]

**Supplementary File 1**

| AccID | FoldChange | Log2FC | P-Value | FDR | Style | 2A_TPM | 4B_TPM |
| --- | --- | --- | --- | --- | --- | --- | --- |
| bta-miR-486 | 2.310836321 | 1.208415 | 0 | 0 | up | 6592.714 | 2852.956 |
| chi-miR-92b | 2.293851219 | 1.197772 | 0 | 0 | up | 13250.56 | 5776.556 |
| chi-miR-92a-3p | 2.202241369 | 1.138973 | 0 | 0 | up | 15935.23 | 7235.913 |
| chi-miR-21-5p | 2.15311881 | 1.106428 | 0 | 0 | up | 25438.88 | 11814.9 |
| chi-miR-223-3p | 88.5702928 | 6.468751 | 1.05E-259 | 2.13E-257 | up | 1109.396 | 12.5256 |
| chi-miR-451-5p | 2.620561756 | 1.389876 | 3.84E-178 | 5.93E-176 | up | 2820.247 | 1076.199 |
| bta-miR-375 | 0.415379831 | -1.2675 | 4.22E-169 | 6.16E-167 | down | 1280.481 | 3082.675 |
| chr22_16643_mature@@ssc-miR-142-5p | 2.280545572 | 1.189379 | 4.82E-125 | 6.66E-123 | up | 2509.738 | 1100.499 |
| chi-miR-93-5p | 2.998733364 | 1.584353 | 1.34E-106 | 1.53E-104 | up | 1379.989 | 460.1905 |
| chr3_2806_mature | 0.387828355 | -1.36651 | 1.37E-104 | 1.50E-102 | down | 654.149 | 1686.697 |
| chr2_1026_mature | 0.418262279 | -1.25752 | 2.75E-104 | 2.89E-102 | down | 799.6788 | 1911.907 |
| bta-miR-2904 | 0.436410204 | -1.19624 | 6.49E-102 | 6.55E-100 | down | 881.6594 | 2020.254 |
| chi-miR-199a-5p | 2.513112602 | 1.329475 | 3.12E-96 | 2.92E-94 | up | 1616.093 | 643.0643 |
| ssc-miR-1285 | 0.465521632 | -1.10308 | 8.62E-92 | 7.81E-90 | down | 962.3962 | 2067.35 |
| chr28_19385_mature | 0.475769645 | -1.07166 | 1.19E-91 | 1.04E-89 | down | 1029.111 | 2163.046 |
| chr19_14868_mature | 0.44677559 | -1.16238 | 4.45E-79 | 3.77E-77 | down | 729.232 | 1632.211 |
| chr2_1153_mature | 0.455083985 | -1.1358 | 8.54E-71 | 7.01E-69 | down | 688.1851 | 1512.216 |
| chr4_3804_mature | 0.433439015 | -1.2061 | 7.16E-69 | 5.70E-67 | down | 579.066 | 1335.98 |
| chi-miR-19b-3p | 2.23096319 | 1.157667 | 7.04E-68 | 5.14E-66 | up | 1404.752 | 629.6619 |
| chi-miR-199a-3p | 2.133683023 | 1.093346 | 9.73E-62 | 6.72E-60 | up | 1396.95 | 654.7131 |
| chi-miR-199c-3p | 2.08419578 | 1.059491 | 2.42E-57 | 1.59E-55 | up | 1362.462 | 653.711 |
| chi-miR-199b-3p | 2.081576835 | 1.057677 | 3.42E-57 | 2.19E-55 | up | 1362.575 | 654.5878 |
| chr7_5852_mature | 0.445183223 | -1.16753 | 5.06E-57 | 3.17E-55 | down | 515.63 | 1158.242 |
| chi-miR-199b-5p | 2.331719972 | 1.221395 | 1.26E-49 | 7.71E-48 | up | 934.0139 | 400.5687 |
| chi-miR-155-5p | 2.39316768 | 1.258921 | 1.66E-48 | 9.88E-47 | up | 869.8994 | 363.4929 |
| chr4_3804_star | 0.481252057 | -1.05514 | 2.15E-48 | 1.25E-46 | down | 553.8499 | 1150.852 |
| chr5_4624_mature | 0.449621822 | -1.15322 | 2.51E-48 | 1.43E-46 | down | 447.5578 | 995.4094 |
| chr16_12774_mature | 0.477977969 | -1.06498 | 7.74E-46 | 4.32E-44 | down | 512.1246 | 1071.44 |
| chr9_7923_mature | 0.418110978 | -1.25804 | 6.62E-45 | 3.55E-43 | down | 336.0641 | 803.7677 |
| chi-miR-199c-5p | 2.227168055 | 1.15521 | 7.49E-45 | 3.93E-43 | up | 918.6355 | 412.468 |
| chi-miR-21-3p | 3.821325058 | 1.934073 | 1.07E-39 | 5.38E-38 | up | 378.1286 | 98.95223 |
| chr7_6470_mature | 0.412143134 | -1.27878 | 5.17E-38 | 2.56E-36 | down | 271.3842 | 658.4707 |
| chr24_17691_mature | 0.339250963 | -1.55958 | 1.41E-35 | 6.48E-34 | down | 154.4628 | 455.3055 |
| chr28_19385_star | 0.448609373 | -1.15647 | 2.55E-34 | 1.16E-32 | down | 310.9611 | 693.1667 |
| chr14_11408_mature | 0.479742111 | -1.05967 | 6.38E-32 | 2.79E-30 | down | 354.8348 | 739.6366 |
| chr5_4652_mature | 0.44200851 | -1.17785 | 1.40E-28 | 5.57E-27 | down | 245.2635 | 554.884 |
| bta-miR-142-3p | 2.620771815 | 1.389992 | 5.16E-28 | 2.02E-26 | up | 419.854 | 160.2024 |
| chr2_1593_mature | 0.44988156 | -1.15238 | 2.30E-26 | 8.75E-25 | down | 237.235 | 527.3277 |
| chr3_2810_mature | 0.431562345 | -1.21236 | 5.36E-26 | 1.96E-24 | down | 206.8174 | 479.2294 |
| chr9_7746_mature | 0.36999105 | -1.43444 | 1.31E-24 | 4.70E-23 | down | 129.0206 | 348.7127 |
| chi-miR-223-5p | 72.13093869 | 6.172546 | 5.76E-23 | 1.96E-21 | up | 90.34832 | 1.25256 |
| chr9_7746_star | 0.481226072 | -1.05521 | 1.37E-22 | 4.61E-21 | down | 247.9773 | 515.3032 |
| chi-miR-17-5p | 3.173085569 | 1.665886 | 2.20E-21 | 7.33E-20 | up | 240.8535 | 75.90513 |
| chr3_2716_mature | 0.424194704 | -1.2372 | 2.72E-20 | 8.82E-19 | down | 150.8444 | 355.6018 |
| chi-miR-20a-5p | 3.043402009 | 1.605685 | 3.66E-20 | 1.17E-18 | up | 238.2527 | 78.28499 |
| chi-miR-301a-3p | 2.658141949 | 1.410418 | 4.18E-20 | 1.32E-18 | up | 287.6673 | 108.2212 |
| chr14_11288_mature | 0.477431375 | -1.06663 | 1.10E-19 | 3.46E-18 | down | 208.2874 | 436.2666 |
| bta-miR-2887 | 0.405360715 | -1.30272 | 4.28E-19 | 1.32E-17 | down | 124.4975 | 307.1277 |
| chr4_3752_mature | 0.395922618 | -1.33671 | 5.30E-19 | 1.62E-17 | down | 116.2429 | 293.6 |
| chr6_5650_mature | 0.461710326 | -1.11494 | 3.55E-18 | 1.07E-16 | down | 171.8767 | 372.2608 |
| chr3_2486_mature | 0.064014259 | -3.96546 | 4.88E-16 | 1.42E-14 | down | 4.409993 | 68.8908 |
| chi-miR-128-3p | 2.238857672 | 1.162763 | 2.80E-15 | 7.98E-14 | up | 287.4411 | 128.3874 |
| chr26_18629_mature | 0.468576323 | -1.09364 | 1.69E-14 | 4.62E-13 | down | 140.2152 | 299.2366 |
| chr10_8225_mature | 0.398309671 | -1.32804 | 3.30E-14 | 8.93E-13 | down | 85.71218 | 215.1898 |
| mmu-miR-6978-5p | 0.456717117 | -1.13063 | 3.94E-14 | 1.06E-12 | down | 125.8544 | 275.5632 |
| chi-miR-19a | 2.610458949 | 1.384303 | 4.97E-14 | 1.32E-12 | up | 199.1282 | 76.2809 |
| chr17_13667_mature | 4.80205821 | 2.263653 | 6.92E-14 | 1.82E-12 | up | 99.84677 | 20.79249 |
| chr12_10061_mature | 0.097457606 | -3.35908 | 7.38E-14 | 1.92E-12 | down | 6.445375 | 66.13516 |
| chi-miR-222-3p | 2.447808868 | 1.291491 | 9.24E-14 | 2.38E-12 | up | 216.7681 | 88.55599 |
| chr5_4364_mature | 0.396940151 | -1.33301 | 3.71E-13 | 9.10E-12 | down | 77.90988 | 196.2761 |
| chr17_13668_mature | 4.770942619 | 2.254274 | 3.74E-13 | 9.11E-12 | up | 94.41909 | 19.79045 |
| chi-miR-1388-5p | 2.204732945 | 1.140604 | 6.15E-13 | 1.48E-11 | up | 246.055 | 111.6031 |
| chr4_3031_mature | 0.41487579 | -1.26925 | 1.70E-12 | 3.98E-11 | down | 82.88526 | 199.7833 |
| chr5_4362_mature | 0.428411471 | -1.22293 | 1.01E-11 | 2.25E-10 | down | 84.35526 | 196.9024 |
| chi-miR-106b-5p | 2.05446885 | 1.038765 | 4.51E-11 | 9.87E-10 | up | 239.8358 | 116.7386 |
| chr3_2518_mature | 0.398360837 | -1.32785 | 4.61E-11 | 1.00E-09 | down | 64.56682 | 162.0813 |
| rno-miR-92a-3p | 3.884330224 | 1.957666 | 8.91E-11 | 1.90E-09 | up | 90.00909 | 23.17236 |
| chr2_1037_mature | 0.460404426 | -1.11903 | 3.02E-10 | 6.36E-09 | down | 89.44371 | 194.272 |
| chr1_309_mature | 0.429633248 | -1.21882 | 3.23E-10 | 6.74E-09 | down | 72.59527 | 168.9703 |
| chr5_3880_mature | 0.428830295 | -1.22152 | 3.40E-10 | 7.03E-09 | down | 72.02989 | 167.9683 |
| chi-miR-15b-5p | 2.649468766 | 1.405703 | 5.15E-10 | 1.06E-08 | up | 132.4129 | 49.97714 |
| chi-miR-34a | 2.990409692 | 1.580343 | 9.48E-10 | 1.92E-08 | up | 107.8752 | 36.07373 |
| chi-miR-9-5p | 8.105261381 | 3.018859 | 2.06E-09 | 4.14E-08 | up | 46.7007 | 5.761776 |
| chr29_19896_mature | 0.087182931 | -3.51981 | 2.78E-09 | 5.53E-08 | down | 3.505379 | 40.20717 |
| chr4_3031_star | 0.452748352 | -1.14322 | 3.32E-09 | 6.56E-08 | down | 74.96988 | 165.5884 |
| chi-miR-144-3p | 2.570866749 | 1.362255 | 5.20E-09 | 1.02E-07 | up | 122.6883 | 47.72253 |
| chrx_20120_star | 2.748934518 | 1.458873 | 9.10E-09 | 1.76E-07 | up | 107.0837 | 38.95461 |
| chr19_14640_mature | 0.395459088 | -1.3384 | 3.10E-08 | 5.86E-07 | down | 44.77839 | 113.2314 |
| chr8_7343_mature | 2.793596218 | 1.482124 | 1.17E-07 | 2.16E-06 | up | 88.87833 | 31.81502 |
| rno-miR-185-3p | 0.467826405 | -1.09595 | 1.89E-07 | 3.33E-06 | down | 64.34067 | 137.5311 |
| chr10_8516_mature | 0.433096307 | -1.20724 | 1.96E-07 | 3.43E-06 | down | 50.88454 | 117.4901 |
| hsa-miR-4792 | 0.476795556 | -1.06856 | 2.57E-07 | 4.44E-06 | down | 66.82836 | 140.1615 |
| ssc-miR-4332 | 0.389475262 | -1.3604 | 3.24E-07 | 5.49E-06 | down | 36.63687 | 94.06725 |
| hsa-miR-4508 | 0.407773486 | -1.29416 | 4.09E-07 | 6.80E-06 | down | 40.70763 | 99.82903 |
| bta-miR-574 | 2.800578234 | 1.485725 | 5.70E-07 | 9.36E-06 | up | 78.92757 | 28.1826 |
| chr17_13175_mature | 0.43478371 | -1.20163 | 8.90E-07 | 1.44E-05 | down | 45.90916 | 105.5908 |
| chr1_672_mature | 0.485625413 | -1.04208 | 1.53E-06 | 2.45E-05 | down | 61.73991 | 127.1348 |
| hsa-miR-4700-5p | 0.403665006 | -1.30877 | 1.69E-06 | 2.68E-05 | down | 35.39302 | 87.67919 |
| chr5_4420_mature | 0.385105398 | -1.37667 | 1.71E-06 | 2.70E-05 | down | 31.20918 | 81.04063 |
| chr6_4879_mature | 0.43963658 | -1.18562 | 1.96E-06 | 3.08E-05 | down | 44.43916 | 101.0816 |
| chr14_11098_mature | 0.270359367 | -1.88705 | 3.74E-06 | 5.78E-05 | down | 13.00383 | 48.0983 |
| chr4_3277_mature@@bta-miR-2285ad | 6.296787201 | 2.654616 | 3.96E-06 | 6.08E-05 | up | 31.54841 | 5.01024 |
| chr1_831_mature | 0.460432873 | -1.11894 | 6.28E-06 | 9.54E-05 | down | 46.02224 | 99.95428 |
| chr15_11686_mature | 0.415048392 | -1.26865 | 7.14E-06 | 0.00010714 | down | 33.58379 | 80.91537 |
| chi-miR-130b-3p | 2.654665955 | 1.40853 | 9.31E-06 | 0.00013815 | up | 67.16759 | 25.30171 |
| chr1_680_star@@bta-miR-2285ad | 6.82716175 | 2.771286 | 1.10E-05 | 0.00016114 | up | 27.36457 | 4.008192 |
| chr7_6002_mature | 0.455601124 | -1.13416 | 4.21E-05 | 0.0006014 | down | 36.63687 | 80.41435 |
| chr2_1153_star | 0.483222861 | -1.04924 | 4.50E-05 | 0.00063494 | down | 43.7607 | 90.56008 |
| chr13_10500_star | 0.392506604 | -1.34921 | 4.68E-05 | 0.00065677 | down | 23.74612 | 60.49864 |
| chr9_7837_mature | 0.445887503 | -1.16525 | 7.47E-05 | 0.00103297 | down | 32.1138 | 72.0222 |
| chr22_16690_mature | 0.402742229 | -1.31207 | 9.40E-05 | 0.0012926 | down | 23.40689 | 58.11878 |
| hsa-miR-6087 | 0.483416199 | -1.04866 | 0.0001588 | 0.00212847 | down | 37.54148 | 77.65871 |
| chr19_14548_star | 0.109621487 | -3.1894 | 0.0001654 | 0.00220614 | down | 1.922305 | 17.53584 |
| chr22_16519_mature@@hsa-miR-4492 | 0.406139118 | -1.29995 | 0.0001926 | 0.00255493 | down | 21.82381 | 53.73482 |
| chi-miR-221-5p | 2.302267207 | 1.203055 | 0.0002021 | 0.00266826 | up | 60.26991 | 26.1785 |
| chr2_1593_star | 0.468461937 | -1.094 | 0.0002167 | 0.00284626 | down | 32.5661 | 69.51708 |
| chr16_12993_mature | 0.124293758 | -3.00817 | 0.000273 | 0.00353225 | down | 2.148458 | 17.28533 |
| chr7_6470_star | 0.482232485 | -1.0522 | 0.0003352 | 0.00427408 | down | 33.58379 | 69.64233 |
| mmu-miR-6937-5p | 0.427182596 | -1.22708 | 0.0003649 | 0.00463051 | down | 22.95458 | 53.73482 |
| chr25_18239_mature | 0.444313964 | -1.17035 | 0.0004577 | 0.00575298 | down | 24.87688 | 55.98943 |
| chi-miR-17-3p | 2.127385796 | 1.089082 | 0.0005397 | 0.00668791 | up | 61.2876 | 28.80888 |
| chrx_20374_mature | 0.442825579 | -1.17519 | 0.0006351 | 0.00779608 | down | 23.40689 | 52.85803 |
| chi-miR-144-5p | 2.700306498 | 1.433123 | 0.0007051 | 0.0085359 | up | 38.21994 | 14.15393 |
| mmu-miR-5623-3p | 0.142167746 | -2.81433 | 0.0007295 | 0.00879141 | down | 2.261535 | 15.90751 |
| chr19_15019_mature | 0.111847015 | -3.1604 | 0.0007513 | 0.00901208 | down | 1.583074 | 14.15393 |
| chr13_10489_mature | 0.346173118 | -1.53043 | 0.0008964 | 0.010607 | down | 11.53383 | 33.31809 |
| chi-miR-191-3p | 2.23864894 | 1.162628 | 0.0011757 | 0.01384962 | up | 48.50993 | 21.66929 |
| chr13_10512_mature | 0.479271222 | -1.06109 | 0.0013671 | 0.01603298 | down | 26.23381 | 54.73687 |
| chr2_1946_mature | 0.49260475 | -1.0215 | 0.0018911 | 0.02178876 | down | 27.02534 | 54.86212 |
| chi-miR-18a-5p | 2.636789417 | 1.398782 | 0.0018876 | 0.02184412 | up | 33.35764 | 12.65086 |
| chr15_12204_mature | 0.476715292 | -1.0688 | 0.0022873 | 0.02589927 | down | 23.40689 | 49.10035 |
| bta-miR-677 | 0.410347814 | -1.28508 | 0.0025505 | 0.02827081 | down | 14.69998 | 35.82321 |
| chr19_15307_mature | 0.445844158 | -1.16539 | 0.0028584 | 0.03128726 | down | 18.20536 | 40.83345 |
| chr15_12382_mature | 0.499355624 | -1.00186 | 0.0029535 | 0.03219411 | down | 25.89458 | 51.85598 |
| chr5_4147_mature | 0.307535394 | -1.70118 | 0.0030304 | 0.03289647 | down | 7.010758 | 22.79659 |
| chr22_16408_mature | 0.200614487 | -2.3175 | 0.0036126 | 0.03842223 | down | 2.939995 | 14.65495 |
| chr24_17599_star | 0.416404608 | -1.26394 | 0.003739 | 0.03928954 | down | 14.13459 | 33.94437 |
| chr4_3275_mature | 0.297317877 | -1.74992 | 0.0037718 | 0.03947656 | down | 6.219221 | 20.91775 |
| hsa-miR-6131 | 0.31491809 | -1.66695 | 0.0044993 | 0.04690331 | down | 6.784605 | 21.54403 |
| chr8_6747_mature | 0.466850534 | -1.09897 | 0.0047541 | 0.04936403 | down | 18.77074 | 40.20717 |
| chr22_16723_mature | 0.161547455 | -2.62997 | 0.004817 | 0.04982021 | down | 1.922305 | 11.89932 |

**Supplementary File 2**

**
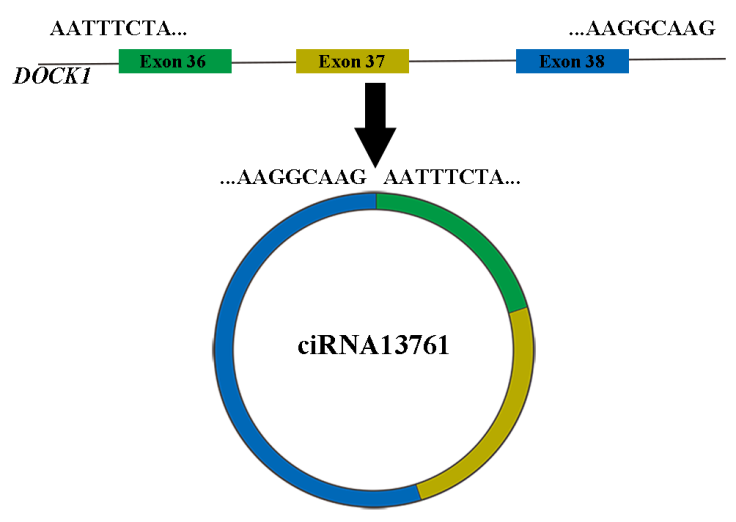
**

**Supplementary File 3**

psiCHECK2-ELF2-1 vector sequencing **result**

GGCGCGGTTGCTAGAATGTTCATCGAGTCCGACCCTGGGTTCTTTTCCACGCTATTGTCGAGGGAGCTAAGAAGTTCCCTAACACCGAGTTCGTGAAGGTGAAGGGCCTCCACTTCAGCCAGGAGGACGCTCCAGATGAAATGGGTAAGTACATCAAGAGCTTCGTGGAGCGCGTGCTGAAGAACGAGCAGTAATTCTAGGCGATCGCTCGAGCTAGGGTGTTAGTGCCGGTCCTGATACGAGTGCTTGCAAGGGGGTCATCGACAGAAGTCAGAAGACTTTGAAACACTTCTAATGCATTTAAGAAAACAATCAAACTTACTGGAAATAAATTACCTATCCCATGTTTCAGTGGGAAATGAACTACATATTGAGATGCTGACAGAAAACTGCCTCTTACAGTAGAAACAACTGAACCCGCCAATAAGGAAAAGGACTGAAAGGGACCAAGCCGCTCACTACAATATCAAGTTACACTAAGACTTGGAACACTACCACTGTAAGAGGTTACACAGTTTTCAGTGGGGAGGGATTGGGATGGGTGATCTTGTCTCACCCCCGTCTCACCTGCATGTACCCAAAAGAGACGTCCAGAGCCAAGGCGGCCGCTGGCCGCAATAAAATATCTTTATTTTCATTACATCTGTGTGTTGATTTTTTGTGTGAGGATCTAAATGAGTCTTCGGACCTCGCGGTGGCCGCTTAAGCGGTGGATAGGGTTTGTCTGACGCGCGTGTAGGGGGAAGGAACGAAACACTCTCATTCGGAGTA

**Supplementary File 4**

psiCHECK2-ELF2-2 vector sequencing result

GGGGGATGCTAGATGTTCATCGAGTCCGACCCTGGGTTCTTTTCCACGCTATTGTCGAGGGAGCTAAGAAGTTCCCTAACACCGAGTTCGTGAAGGTGAAGGGCCTCCACTTCAGCCAGGAGGACGCTCCAGATGAAATGGGTAAGTACATCAAGAGCTTCGTGGAGCGCGTGCTGAAGAACGAGCAGTAATTCTAGGCGATCGCTCGAGTTCCCAACTGCTGCGTGAAGAAATTGTTTTGTTCCATTACATTAATATTTGGCAAATAACCTGCATGTTTCCACTTCCTAATTACTGTCATGTAATTATTATAATGTTCTACTTACCCTAATGAATTTACTATTTTAGTGTTGGCCACACTTAAATAATTTATATTTCAAATCAGACCATTTGTTTCTCAATCAGACCTTTAGTGGGTGAGGAAGAACAAATAATACTCTAGCCCTTTTAGAAACTGGCGGGACCAGAGGAACCTCATTTTACACCAAACTAGGTCCTGTAACTCGCGGCCGCTGGCCGCAATAAAATATCTTTATTTTCATTACATCTGTGTGTTGGTTTTTTGTGTGAGGATCTAAATGAGTCTTCGGACCTCGCGGGGGCCGCTTAAGCGGTGGTTAGGGTTTGTCTGACGCGGGGGGAGGGGGAAGGAACGAAACACTCTCATTCGGAGGCGGCTCGGGGTTTGGTCTTGGTGGCCACGGGCACGCAGAAGAGCGCCGCGATCCTCTTAAGCACCCCCCCGCCCTCCGTGGAGGCGGGGGTTTGGTCGGCGGGTGGTAACTGGCGGGCCGCTGACTCGGGCGGGTCGCGCGCCCCAGAGTGTGACCTTTTCCGGCTGGTCGCAGACCCCCGGACG

**Supplementary File 5**

pcDNA3.1(+)-ELF2 sequencing result

GGCGTAGGGCTAATAGCAGAGGCTCTCTGGCTACTAGAGAACCACTGCTTACTGGCTTATCGAAATTAATACGACTCACTATAGGGAGACCCAAGCTGGCTAGCGTTTAAACTTAAGCTTGGTACCCATTTCTCACACGCTACCAGTCCTTCCGTTTTCATTGTCACAGGAAGGGCAAGAGCTGAAGGCGCACTGACAACCACTACGTGGGTCACTGTCTTATTTCCATCTGCCGGTTTGTCGTCTTGTACCAGCTGCAAAGTTTTCACATCATGTTCCTGCTTTTTCGCCACTGCTTCTGATTTAACCTCTGGCCCTTTTATGACTGCGCTGATAACCCGGGGAGGAGTCTGGCCAGATGCCTGCTGAGGAGGCACTGATAGTCTCATCACAGGGGCCCCGTGAGCTATTGAAACGGGGGTAAGTGCTCTCACAGCCAGTGGGGTGCCCACAATGTTAATGCTTCCTGATCCAGTCAGATTCGACTTTGTCTGCAGTTGACACTGTGCAAGTTGCGTGGCTGGGATGGTAATAATTTTGGCAGGCTGCATGGTGATTTTGTCTCCATTTTCAGTAGAGGCTGGCATCACAGTAGGGATTGTCTGAATAACTACCTTTGGAGAGGCTGCTGTTGTCGGACTAGAGCTGGTTATTAACGGTGCACCTGCATTAACTGACTGAACCGCCACAGTCGAAATTTTCTGGCCCAACGATGTCATTACAACAGGTACTTGCATTGCCACACGAACTGTCCTTGGGGCTGCTGCTGCTGATACAGATGTGGTAGTAGTGGGAGACCTGGGTGAAGTGTCATGACCAGGGGGAGTGATGTTCACAACTCTAGCTACACCCTTCTCTGCTCTGGAGCAGTTTAGAGGAGATGAGTTTTTCCCACCACGAACAGAAGTTGCTGCTTTTAAGAGACTTTCTGCAGACAATGATACTCGTTCTAATGATTTTTCATCCGTAGCTGCTGCTAAATCTTCACTACAGGTTTCACTTTTGGCATCATCTATGACCACTATGTTTTTGGGCATATCCTTGAACTGAAATACAAGCCCTCTGTCCTTCAACCTTTGCAAGGATACCCCTTTGGTAATAATATCTCAAAGCTCGTCCCATAGTTTCATAATTCATGTCTGGCTTGTTCTTATGCTTTCCCCAAAGCTTAGAGACAGCCTTTGAATCCACAAGCTTGAATATGCCTTTTTCTCTCTGAGTCCACTTAATGTATCGAGGACAAGTATTTTTGTCTTGAAGTAGATCTAAAAGAAACTCCCACAAATAGGTTGTGTTTCCTTTTCCTTCTCTGGGTTTCTTCTTTATACCTAAGTCAGGAGACCCATTGGAAATTGGCGACTGCTGGGTCTTTGGTTTACGGCCAACTTTTTTCTTTTTCATTGGTTCATGGCTATCCGGTGATGTAGGAATAGGAGAGGTATCCATTGGTTCAGATTCTTCAGTTGACACCTCCACTACAGTTTCTGTAATGACATCTGGCCTCATAGCAGCATGGATAAATTCAGGACTTCTTGAATCTCTCAAGCAGGTAGGAGATTCCATATGAAGCAGGGCTTCAGCAGCTTCAATTGTCTTATCTGTACAGTGTGCATTACTGCTATGAACTGATGCTTCCACAGCCCGGATGAGCAGATCCAGCTGGTTCGTGGGTCCTCATGCAGAGACGTCGCATGGTGGCAAGCTTGGGCGCTTTCCAGTCGGGAAACCTGGCGTGCAACTTGCTTAA

**Supplementary File 6**

| ID | Gene ID | FPKM_  sample1 | FPKM_  sample2 | log2FoldChange | test_stat | p_value | padj | significant |
| --- | --- | --- | --- | --- | --- | --- | --- | --- |
| MRPL57 | gene12699 | 149.5 | 53.4 | -1.5 | -13 | 5.00E-05 | 0.0002 | Down |
| UBE2S | gene18487 | 139 | 67.9 | -1 | -20 | 5.00E-05 | 0.0002 | Down |
| CEBPD | gene14272 | 71.35 | 25 | -1.5 | -21 | 5.00E-05 | 0.0002 | Down |
| JUND | gene8509 | 62.24 | 28.4 | -1.1 | -18 | 5.00E-05 | 0.0002 | Down |
| ISG15 | gene16221 | 50.31 | 7.76 | -2.7 | -12 | 5.00E-05 | 0.0002 | Down |
| LOC108634705 | gene27673 | 46.44 | 21.7 | -1.1 | -9.1 | 5.00E-05 | 0.0002 | Down |
| IFI6 | gene1279 | 46.08 | 14.4 | -1.7 | -10 | 5.00E-05 | 0.0002 | Down |
| C6H4orf48 | gene7028 | 12.48 | 1.06 | -3.6 | -3.6 | 0.00085 | 0.0031 | Down |
| CEBPB | gene13827 | 28.68 | 12.7 | -1.2 | -13 | 5.00E-05 | 0.0002 | Down |
| LOC102190983 | gene16737 | 28.2 | 7.29 | -2 | -13 | 5.00E-05 | 0.0002 | Down |
| MX1 | gene915 | 23.81 | 5.99 | -2 | -18 | 5.00E-05 | 0.0002 | Down |
| LOC102168687 | gene24636 | 19.13 | 3.38 | -2.5 | -21 | 5.00E-05 | 0.0002 | Down |
| ANKRD9 | gene21222 | 14.31 | 3.35 | -2.1 | -11 | 5.00E-05 | 0.0002 | Down |
| MX2 | gene914 | 12.66 | 2.49 | -2.3 | -15 | 5.00E-05 | 0.0002 | Down |
| C6H4orf48 | gene7028 | 12.48 | 1.06 | -3.6 | -3.6 | 0.00085 | 0.0031 | Down |
| CAMK2G | gene25134 | 11.79 | 5.53 | -1.1 | -1.8 | 0.01525 | 0.0375 | Down |
| LOC108638215 | gene19824 | 10.94 | 1.1 | -3.3 | -5.7 | 5.00E-05 | 0.0002 | Down |
| ZNF444 | gene18566 | 10 | 3.3 | -1.6 | -8 | 5.00E-05 | 0.0002 | Down |
| DDX58 | gene8711 | 9.914 | 2.94 | -1.8 | -13 | 5.00E-05 | 0.0002 | Down |
| TMEM238 | gene18489 | 9.616 | 4.64 | -1.1 | -6.4 | 5.00E-05 | 0.0002 | Down |
| LOC102182233 | gene10300 | 9.19 | 4.55 | -1 | -5.8 | 5.00E-05 | 0.0002 | Down |
| MAF | gene17233 | 8.84 | 4.37 | -1 | -6.7 | 5.00E-05 | 0.0002 | Down |
| IFIT3 | gene24638 | 8.252 | 2.81 | -1.6 | -8.6 | 5.00E-05 | 0.0002 | Down |
| EPSTI1 | gene12897 | 7.041 | 2.82 | -1.3 | -6 | 5.00E-05 | 0.0002 | Down |
| PPP1R3G | gene22073 | 6.569 | 12.7 | 0.96 | 12.6 | 5.00E-05 | 0.0002 | Ups |
| PCSK1N | gene27905 | 6.559 | 2.93 | -1.2 | -4.2 | 5.00E-05 | 0.0002 | Down |
| LOC108634568 | gene27379 | 6.287 | 0.89 | -2.8 | -6 | 5.00E-05 | 0.0002 | Down |
| C20H5orf38 | gene20517 | 5.438 | 1.49 | -1.9 | -3.6 | 0.0002 | 0.0008 | Down |
| ZNF580 | gene18471 | 4.901 | 0.79 | -2.6 | -5.4 | 5.00E-05 | 0.0002 | Down |
| ELF2 | gene17037 | 4.205 | 2.83 | -0.6 | -11 | 5.00E-05 | 0.0002 | Down |
| FHL1 | gene26935 | 3.92 | 0 | #### | #### | 5.00E-05 | 0.0002 | Down |
| TOR4A | gene12466 | 3.339 | 1.14 | -1.5 | -4.4 | 5.00E-05 | 0.0002 | Down |
| IFI44L | gene2805 | 3.249 | 0.7 | -2.2 | -7.1 | 5.00E-05 | 0.0002 | Down |
| ZNF579 | gene18477 | 3.128 | 1 | -1.6 | -5.2 | 5.00E-05 | 0.0002 | Down |
| LOC108636851 | gene10197 | 2.804 | 0.41 | -2.8 | -3.6 | 0.00135 | 0.0047 | Down |
| LOC102189040 | gene10660 | 2.676 | 1.16 | -1.2 | -1.7 | 0.01615 | 0.0394 | Down |
| LOC108634838 | gene27999 | 2.257 | 0 | #### | #### | 0.0078 | 0.0213 | Down |
| LOC108634820 | gene27933 | 1.976 | 0.85 | -1.2 | -2.4 | 0.0021 | 0.0069 | Down |
| LOC108635462 | gene28776 | 1.91 | 0 | #### | #### | 5.00E-05 | 0.0002 | Down |
| LOC108636404 | gene8477 | 1.892 | 0.46 | -2 | -5.3 | 5.00E-05 | 0.0002 | Down |
| IFIT2 | gene24639 | 1.818 | 0.24 | -2.9 | -7.2 | 5.00E-05 | 0.0002 | Down |
| FAM174A | gene7116 | 1.544 | 0.6 | -1.4 | -1.8 | 0.01635 | 0.0398 | Down |
| LOC102169889 | gene15219 | 1.529 | 0.54 | -1.5 | -4.4 | 5.00E-05 | 0.0002 | Down |
| MISP3 | gene8225 | 1.502 | 0.66 | -1.2 | -2.3 | 0.0065 | 0.0182 | Down |
| IFI44 | gene2806 | 1.159 | 0.29 | -2 | -3 | 0.0005 | 0.0019 | Down |
| SOCS1 | gene23608 | 1.141 | 0.2 | -2.5 | -2.4 | 0.01715 | 0.0415 | Down |
| FSBP | gene13937 | 1.079 | 0.4 | -1.4 | -2.1 | 0.0037 | 0.0112 | Down |
| LOC108634655 | gene27557 | 1.066 | 0 | #### | #### | 0.00095 | 0.0034 | Down |
| PSCA | gene27512 | 0.994 | 2.1 | 1.08 | 2.72 | 0.00085 | 0.0031 | Ups |
| FHIT | gene21573 | 0.917 | 0.21 | -2.1 | -1.3 | 0.02135 | 0.0499 | Down |
| SH3GL3 | gene20742 | 0.913 | 0.45 | -1 | -1.5 | 0.0133 | 0.0333 | Down |
| LOC108633328 | gene18307 | 0.907 | 0.11 | -3.1 | -3 | 0.00835 | 0.0226 | Down |
| RTP4 | gene463 | 0.892 | 0.12 | -2.9 | -3 | 0.00965 | 0.0255 | Down |
| TMEM163 | gene1788 | 0.823 | 0.4 | -1 | -1.7 | 0.01835 | 0.0439 | Down |
| TNFSF13B | gene12510 | 0.679 | 0.17 | -2 | -1.9 | 0.0012 | 0.0042 | Down |
| LOC108634770 | gene27822 | 0.649 | 0.27 | -1.3 | -2.5 | 0.00415 | 0.0124 | Down |
| MRLN | gene25271 | 0.606 | 0 | #### | #### | 0.0078 | 0.0213 | Down |
| LOC100860781 | gene25538 | 0.606 | 0 | #### | #### | 0.0078 | 0.0213 | Down |
| NEIL1 | gene20860 | 0.48 | 1.24 | 1.37 | 1.92 | 0.0018 | 0.006 | Ups |
| AK8 | gene12363 | 0.456 | 1.22 | 1.43 | 2.1 | 0.0027 | 0.0085 | Ups |
| LOC108634775 | gene27829 | 0.331 | 2.11 | 2.67 | 6.49 | 5.00E-05 | 0.0002 | Ups |
| LOC108635081 | gene28312 | 0.309 | 2.56 | 3.05 | 5.28 | 5.00E-05 | 0.0002 | Ups |
| TSPAN15 | gene25202 | 0.293 | 0.69 | 1.24 | 1.53 | 0.02095 | 0.0491 | Ups |
| EFHC1 | gene22625 | 0.29 | 0.79 | 1.45 | 2.33 | 0.00345 | 0.0106 | Ups |
| LOC108634846 | gene28011 | 0.033 | 1.1 | 5.08 | 2.64 | 0.0167 | 0.0406 | Ups |
| NPB | gene19886 | 0 | 0.64 | inf | #### | 0.00105 | 0.0037 | Ups |
| TCAP | gene19512 | 0 | 0.81 | inf | #### | 0.00025 | 0.001 | Ups |

**Supplementary File 7**

**
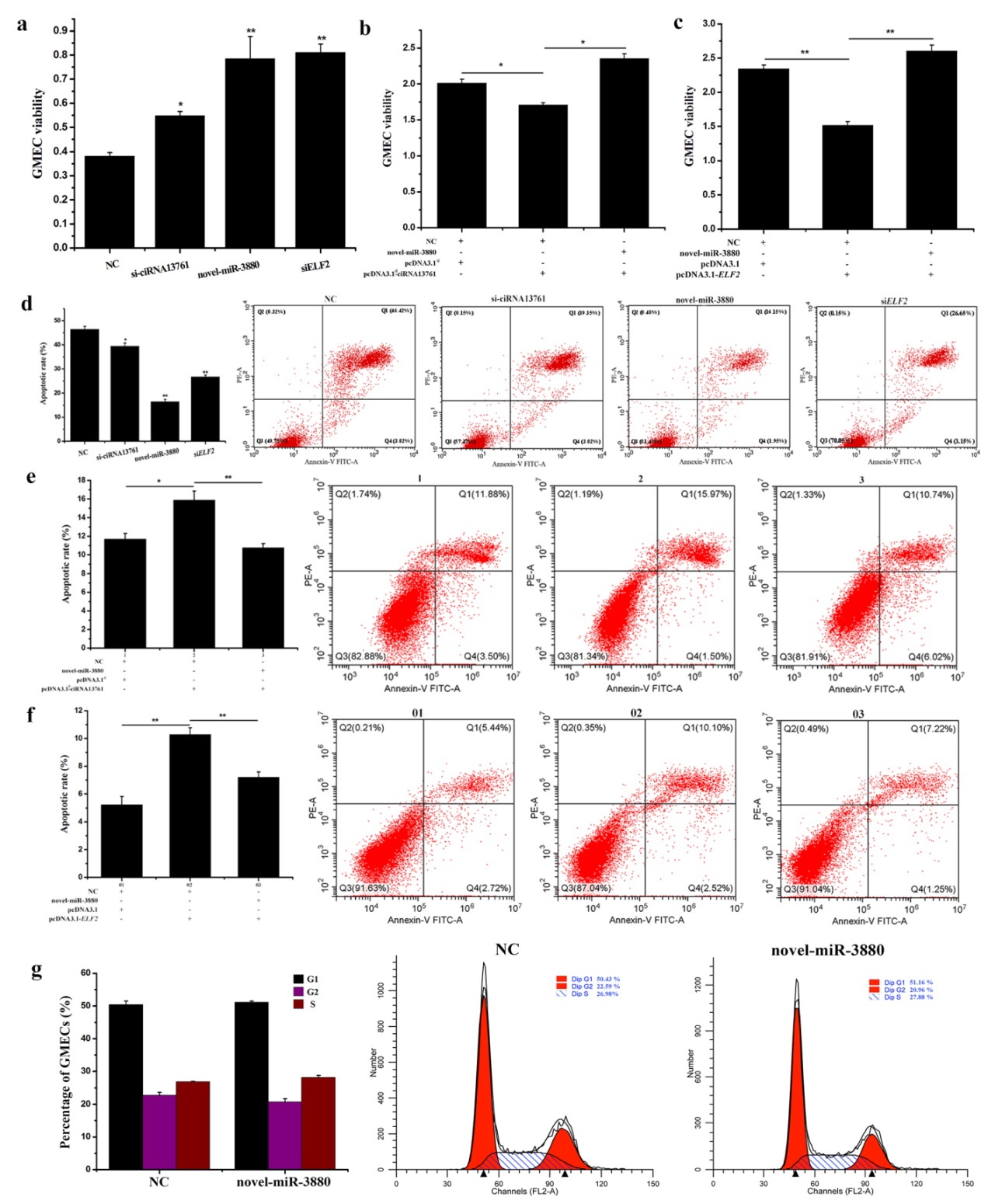
**

**Supplementary File 8**

**
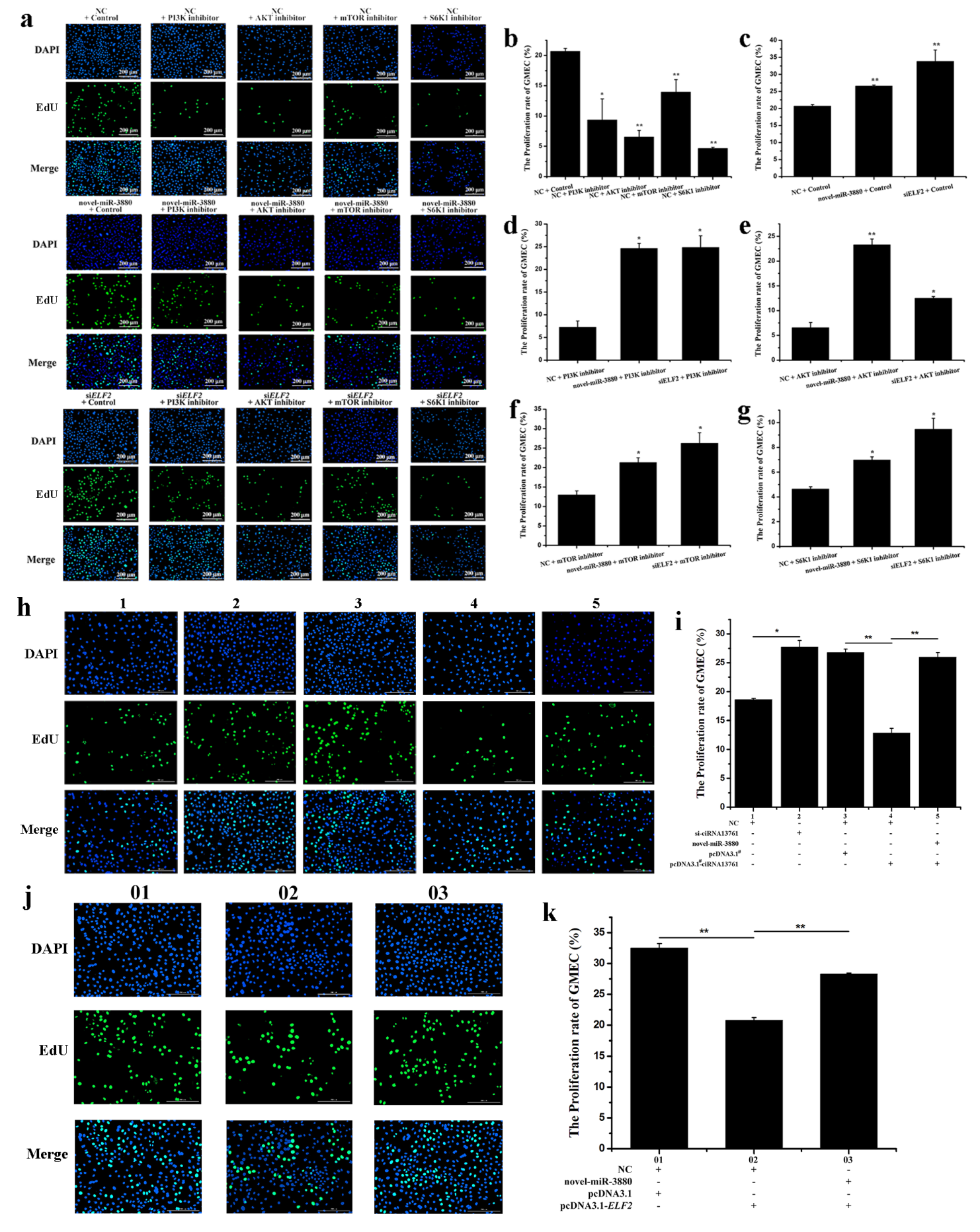
**

**Supplementary File 9**

**
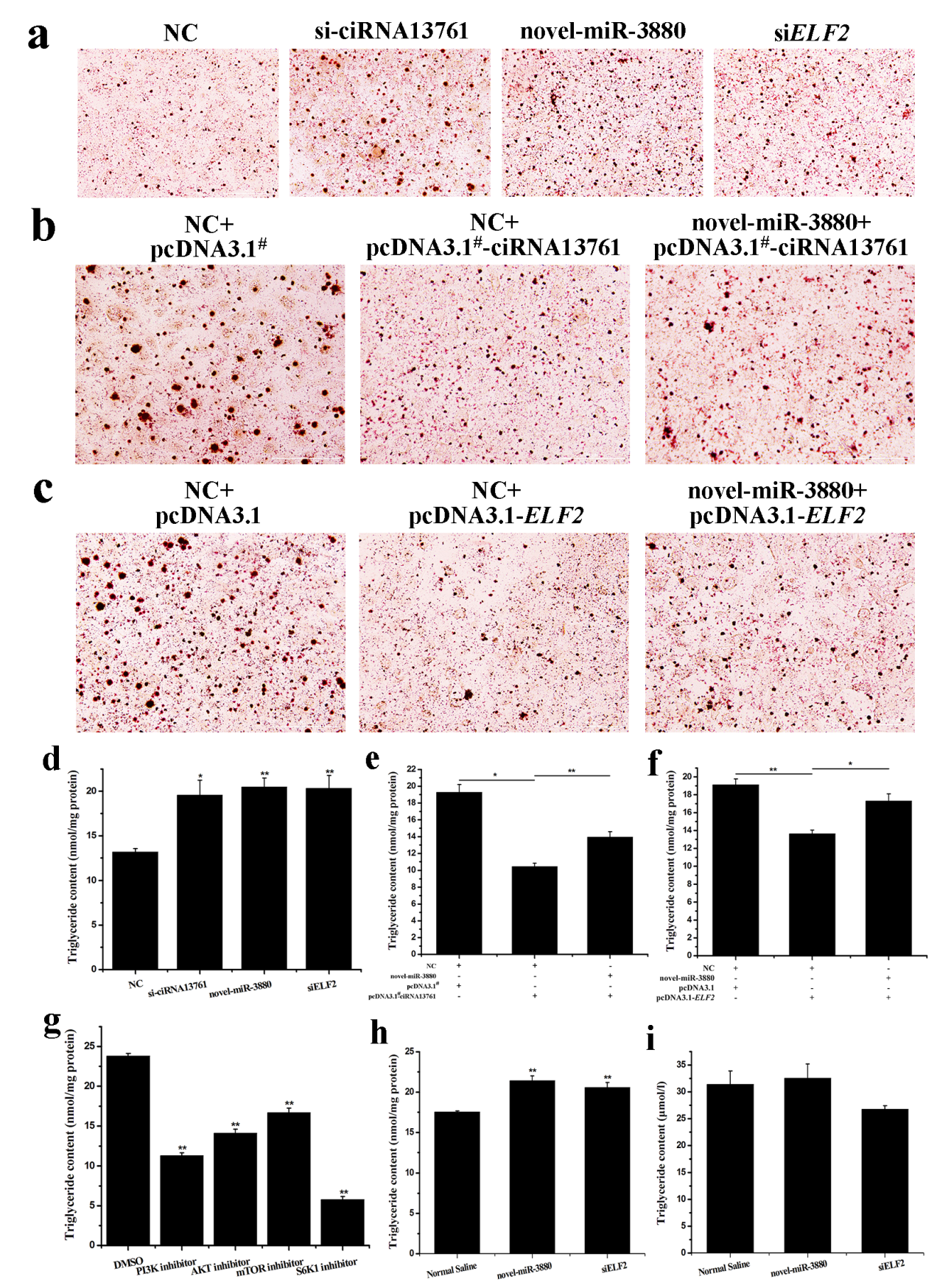
**

**Supplementary File 10**

**
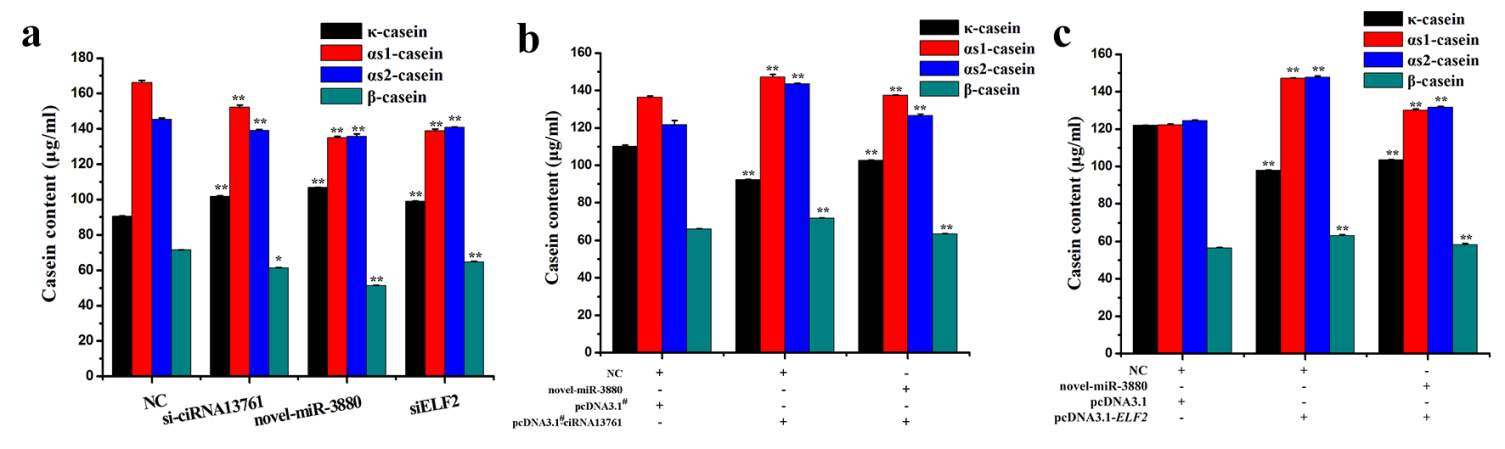
**

**Supplementary File 11**

**
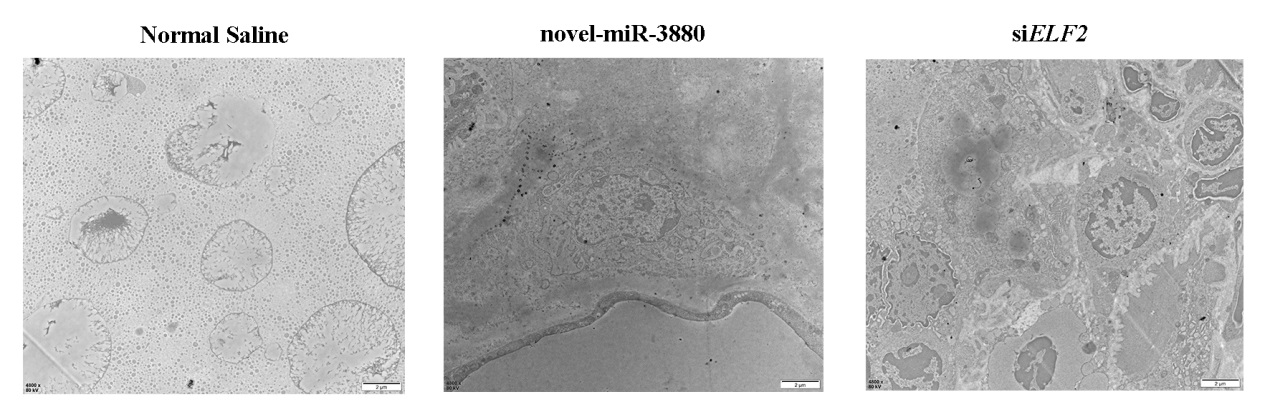
**

**Supplementary File 12**

**
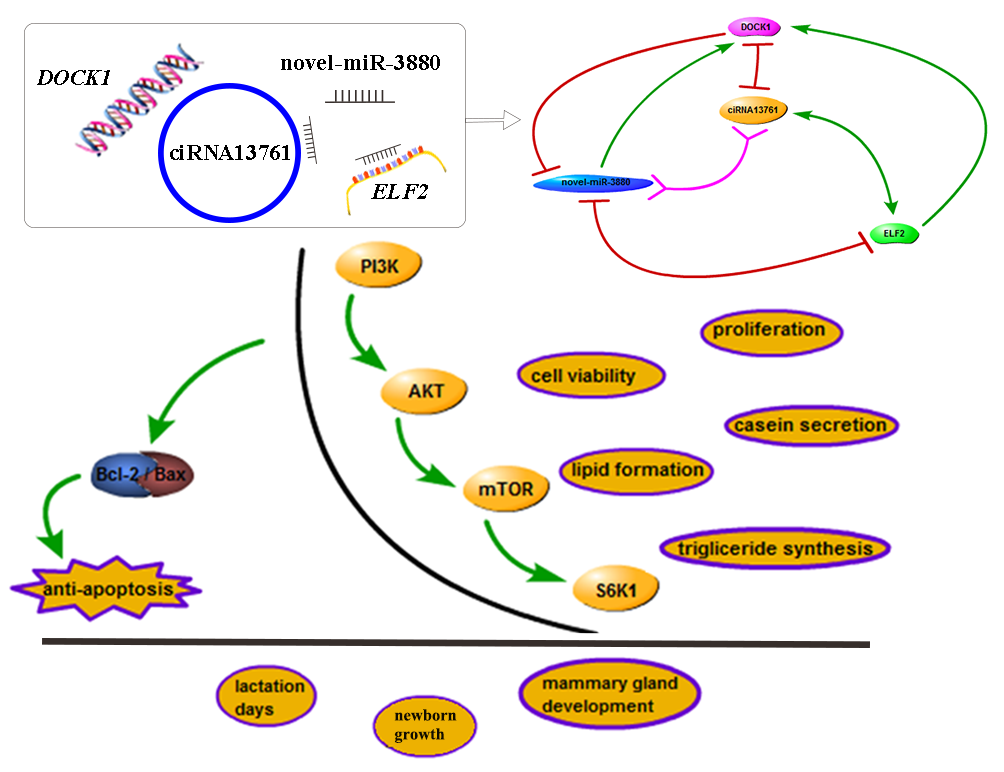
**
